# Interaction between Ni and HZSM-5 in aromatization-enhanced reactive adsorption desulfurization catalysts for FCC gasoline upgrading

**DOI:** 10.1007/s13203-014-0072-z

**Published:** 2014-07-19

**Authors:** Jinchong Zhao, Lulu Zhang, Nannan She, Yunqi Liu, Yongming Chai, Chenguang Liu

**Affiliations:** State Key Laboratory of Heavy Oil Processing, Key Laboratory of Catalysis CNPC, China University of Petroleum, Qingdao, 266580 Shandong China

**Keywords:** Gasoline, Reactive adsorption desulfurization, Aromatization, Coupling, Interaction

## Abstract

A compound catalyst (RA) consisted of Ni, ZnO and HZSM-5 with functions of reactive adsorption desulfurization (RADS) and olefin aromatization for fluid catalytic cracking (FCC) gasoline upgrading was prepared. X-ray powder diffraction (XRD), temperature-programmed reduction and low-temperature N_2_ adsorption were used to characterize the properties of the catalysts. Performance evaluation by FCC gasoline was carried out, and the result showed that the catalyst RA performed well in desulfurization and aromatization. For comparison, RADS catalyst (represented by DS) consisted of Ni and ZnO and aromatization catalyst (represented by Ar) consisted of HZSM-5 were prepared, respectively. They were combined in different ways to help investigating interaction between Ni and HZSM-5. Performance evaluated by FCC gasoline showed that catalyst RA performed best in desulfurization with a slight octane number loss. Interaction between Ni and HZSM-5 is a significant factor which influences the performance of the catalyst.

## Introduction

In the light of increasingly serious air pollution caused by the use of fossil fuel, sulfur in fuels, especially gasoline and diesel, has been a hot issue of refining industry for several years. Many countries have set the content of total sulfur in gasoline and diesel to lower than 10 μg g^−1^ in their regulations [1]. It means that the technique of ultra-deep desulfurization of oil must be updated to make fuels meet rigorous standards.

Fluid catalytic cracking (FCC) gasoline is an important component of commercial gasoline, which contribute the most sulfur emission, up to 80–90 % [2]. On the other hand, a large percentage of olefins in FCC gasoline, which contribute a considerably high octane number, are restricted strictly. So people have to decrease the content of sulfur and olefins in FCC gasoline, while the octane number must be preserved.

Traditional hydro-desulfurization (HDS) has been a significant approach for FCC gasoline desulfurization for many years. However, it takes a large quantity of olefins in FCC gasoline to be saturated during the hydrogenation process, which leads to a great decrease of octane number [3]. In order to avoid that, many non-HDS approaches have been researched to remove sulfur from FCC gasoline, such as catalytic distillation [4], extraction [5], oxidation [6] and adsorption [7]. Remarkably, reactive adsorption desulfurization (RADS) technique is a good choice for FCC gasoline upgrading. A representative process is S-Zorb, which was developed by Conoco Philips Company. Catalysts of S-Zorb are consisted of Ni, ZnO, SiO_2_ and Al_2_O_3_. Organic sulfides are easy to transform to inorganic ones on active Ni species, while ZnO can adsorb inorganic sulfides well. A mechanism called auto-regeneration which explains the whole processing of reactive adsorption desulfurization has been concluded [3].

Although the reaction performs under low H_2_ partial pressure, it cannot completely avoid olefins being saturated [8]. So, some measures for recovering the octane number have been taken following the desulfurization process for FCC gasoline, such as isomerization [9], alkylation [10] and aromatization [11]. Aromatization has been used for FCC gasoline upgrading since 1980s [12]. The excess olefins in FCC gasoline can be transformed to aromatics, which compensates a significant octane number loss. It takes the advantage of acidity and shape-selectivity of HZSM-5 zeolites to produce alkylated aromatics rather than benzene.

Nowadays, many HDS technologies have been combined with octane recovery processing, but most of them are consisted of two separated units, leading to complicated processes and high cost. A one-step reaction with compound catalyst has been a new approach for solving the problems. Our precious study [13] has prepared a catalyst with function of reactive adsorption desulfurization coupling aromatization (RADS-Ar), in which HZSM-5 Zeolite was introduced into the RADS catalyst to form a bifunctional catalyst. The catalyst showed good performances of desulfurization and aromatization, but some further studies should be performed to explore the compound catalytic system.

In this work, a compound catalyst (represented by RA) consisted of Ni, ZnO and HZSM-5 was prepared, and the catalytic performance was evaluated by FCC gasoline on a fixed-bed reactor. The interaction between Ni species and HZSM-5 of the compound system was investigated. For comparison, RADS catalyst (represented by DS) consisted of Ni and ZnO and aromatization catalyst (represented by Ar) consisted of HZSM-5 were prepared, respectively. They were filled in the reactor in different methods to form different bed-filling types, so that DS and Ar could have different distance in the catalytic system. Relationship between Ni species and HZSM-5 was studied.

## Experiment

FCC gasoline from Changqing Petrochemical, CNPC was used as feedstock to test the desulfurization and aromatization performance of the compound system. Table 1 shows its major properties.

**Table 1:** Major properties of Changqing FCC gasoline

Item	Data
Density (20 °C)/g cm^−3^	0.7245
RON	86.3
Total sulfur/μg g^−1^	110
Hydrocarbon group composition/wt%	
n-paraffins	7.09
Isoparaffins	33.52
Olefins	30.36
Naphthenes	6.52
Aromatics	21.71
Boiling range/°C	
IBP/10 %	34.8/49.6
20 %/30 %	60.2/74.4
50 %	111.8
70 %/80 %	149.2/164.9
90 %/FBP	182.9/211.9

The RA catalyst is a compound catalyst, which consists of RADS part based on Ni/ZnO and aromatization part based on HZSM-5. ZnO, HZSM-5, silicasol and Al_2_O_3_ were mixed uniformly and extruded. After drying and calcination, Ni species were introduced by incipient wetness impregnation. The RA catalyst was finally obtained after calcination at 673 K.

The DS catalyst was prepared by extruding the mixture of ZnO, silicasol and Al_2_O_3_, and followed with introduction of Ni species by impregnation. The Ar catalyst was prepared by extruding the mixture of HZSM-5, silicasol and Al_2_O_3_. The DS catalyst and the Ar catalyst needed calcination at 673 K before they could be used. After the DS and the Ar were finally obtained, they would be combined in different ways for the following evaluations.

**Fig. 1:**
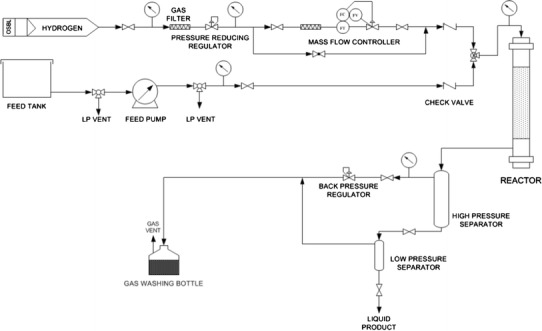
Scheme of 100 mL pilot fixed-bed reactor system

**Fig. 2:**
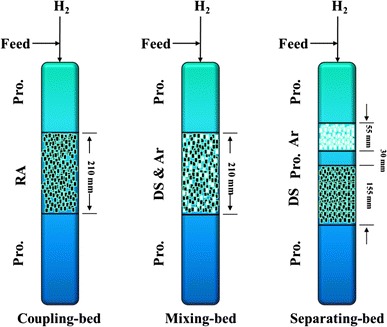
Scheme of different catalyst bed-filling types. *RA* reactive adsorption desulfurization coupling aromatization catalyst, *DS* reactive adsorption desulfurization catalyst, *Ar* aromatization catalyst, *Pro* protective catalyst

Performance evaluations of catalysts were carried out in a fixed-bed reactor, which could hold 100 cm^3^ of the catalysts (Fig. 1). The RA catalyst was filled in an independent reactor. For comparison, the DS catalyst and the Ar catalyst were filled in the reactor after being mixed together or separated by SiO_2_, respectively. Figure 2 shows three reactors filled by different methods, and they were named as coupling-bed, mixing-bed and separating-bed according to the combination states.

Before the catalysts works, Ni species in the catalysts must be reduced to reduced state (Ni^0^) under 633 K and 0.5 MPa with a H_2_ flow at a rate of 20 L/h. Then the FCC gasoline was fed by a ram pump, and the reaction was performed at 673 K, 0.5 MPa, a H_2_/feed volumetric ratio of 200/1, and LHSV of 1.0 h^−1^. Outlet products were collected for analysis. Sulfur content was analyzed by a Multi EA 3100 S/N Analyzer and the hydrocarbon group composition analyzed by Agilent 7890A gas chromatograph.

## Results and discussion

**Fig. 3:**
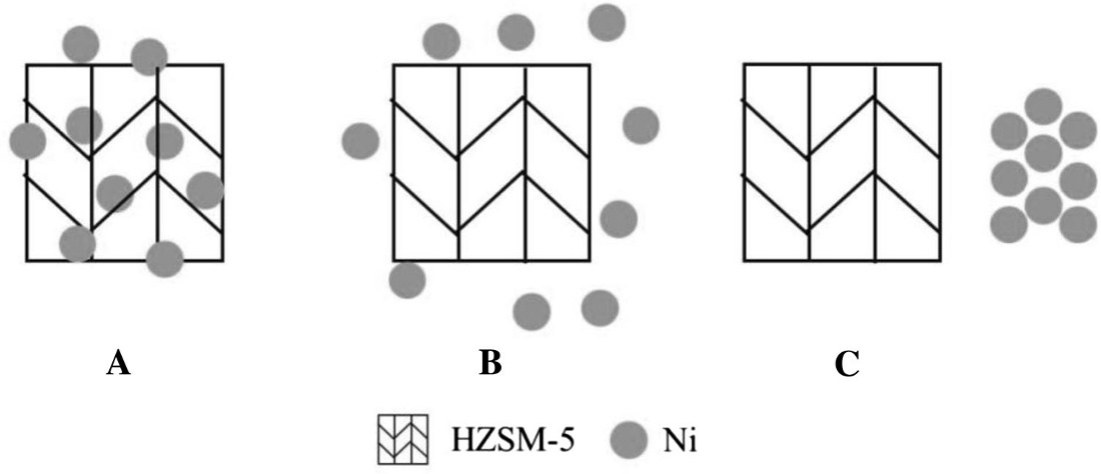
Scheme of microscopical contact types between Ni and HZSM-5

Nickel is a kind of sulphophile element, which has an excellent sulfur adsorption property. It also has good adsorption and hydrogenation abilities to olefins for unoccupied d orbitals [14, 15]. HZSM-5 zeolite, which has a particular 2-dimension pore structure, consists of Si–O tetrahedrons and Al–O tetrahedrons. It can be used as an active component for desulfurization and aromatization because of the strong acidity, but the performance of desulfurization has been proved to be not very good [16, 17]. In the catalytic system of RADS, Ni plays a role of freights, which can catch sulfur species and olefins. A coupling system can be formed by Introducing the HZSM-5, which can supply acid sites and appropriate pores for organic sulfides cracking and olefin aromatization [18]. Figure 3 shows three different relationships between Ni species and HZSM-5 in corresponding combination states. In coupling-bed, Ni species in RA catalyst are introduced by impregnation, so that Ni species can deposit on the surface or into the pores of HZSM-5, this is a significant precondition of forming a closely contact system. In mixing-bed and separating-bed, Ni species cannot diffuse into the inside space of HZSM-5, they can only get in touch or keep a certain distance with each other as shown in Fig. 3.

**Fig. 4:**
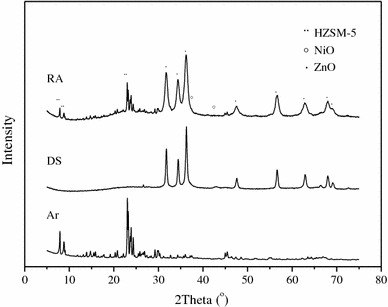
XRD pattern of the catalysts. *RA* reactive adsorption desulfurization coupling aromatization catalyst, *DS* reactive adsorption desulfurization catalyst, *Ar* aromatization catalyst

Figure 4 is X-ray powder diffraction patterns of three different catalysts. The main active component of Ar catalyst is HZSM-5, it shows relatively high-intensity peaks that represent HZSM-5 (ICDD/PDF2 card 00-049-0657). DS catalyst is prepared mainly by ZnO, with Ni species being impregnated on the support, so peaks represent ZnO (ICDD/PDF2 card 01-079-2205) are very strong while peaks of NiO (ICDD/PDF2 card 03-065-2901) are very weak. In RA catalyst, two active parts are coupled in one system, Ni species are impregnated on the support, which is prepared by ZnO and HZSM-5. Ni species can be uniformly dispersed after calcination, so peaks represent NiO can hardly be found in the X-ray powder diffraction (XRD) pattern of RA catalyst. Uniformly dispersed Ni species can contact with HZSM-5 closely as the scheme shown in Fig. 3, it is very easy for them to form a compound system with interaction.

Pore structure and specific surface areas of different catalysts according to the BET method were determined. Table 2 shows the data about the textural properties.

**Table 2:** Textural properties of different catalysts

Catalyst	*S*_BET_ (m^2^/g)	*S*_micro_ (m^2^/g)	*S*_meso_ (m^2^/g)	*V*_meso_ (cm^3^/g)	*D*_meso_ (nm)	*V*_micro_ (cm^3^/g)	*D*_micro_ (nm)
DS	85	13	72	0.25	10.17	0.02	0.68
AR	130	67	63	0.25	11.98	0.04	0.55

**Fig. 5:**
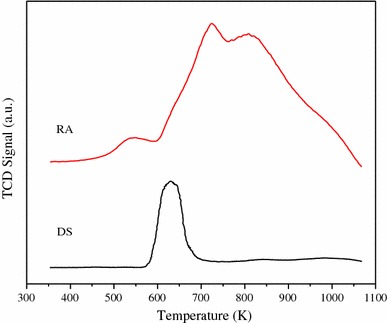
TPR profiles of RA and DS

Ni species are needed to be reduced before reaction, so the reduction temperature was determined by temperature-programmed reduction (TPR). Figure 5 shows the TPR profiles of RA and DS catalysts. For DS catalyst, only one narrow NiO reduction peak at 633 K is observed, which implies that the interaction between NiO and the support is weaker. For RA catalyst, besides a low-temperature peak at about 540 K ascribes to the reduction of NiO on the surface, another broad peak at higher temperature area, which could be attributed to the reduction of NiO contacted with the support.

**Fig. 6:**
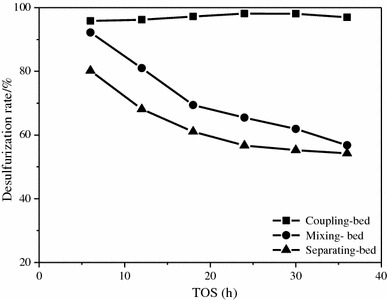
Desulfurization rate curve over different bed-filling types

The desulfurization rate is calculated according to the sulfur content in feed and products, so we get a series of data to show the desulfurization performances of different compound systems. Figure 6 shows desulfurization rate of different compound systems, they all change with time in a certain rule. Curve representing the coupling-bed indicates the trend of desulfurization rate of RA catalyst. The desulfurization rate reaches 95 % at the beginning, and the curve keeps in a relatively high level in the following 36 h, with no sharp falling happening in the end. Other curves indicate the desulfurization rate of mixing-bed and separating-bed, respectively. The desulfurization rate of mixing bed reaches 92 %, while the separating-bed only reaches 80 % at the beginning. Although they achieve a high performance initially, a great deterioration occurs soon. During the following process, curves representing mixing-bed and separating-bed drop sharply, the desulfurization rate of mixing-bed drops to around 60 % in the following 36 h, and the other curve have a faster decrease to 60 % in 18 h.

Some research have shown that nano-scaled HZSM-5 has a function of desulfurization [19]. Thiophene molecules can be adsorbed by HZSM-5, and acid sites of HZSM-5 can improve the cracking of the C–S bond of thiophene molecules. The cracking products are usually H_2_S or quasi-mercaptan, which are easy to be removed. In addition, Ni species can effectively enhance the desulfurization activity and stability of HZSM-5 [13].

Here, the trend shown in Fig. 6 is caused by different existing states of the active element Ni, which is introduced into the catalytic system by different methods. In the view of space, the distance between Ni species and HZSM-5 in three different states can be coupling-bed < mixing-bed < separating-bed. HZSM-5 has a function of desulfurization itself which is not very perfect. However, the existing of Ni species can help it perform better in the compound systems, because Ni species and HZSM-5 can form an interaction couple to enhance the performance. The closer distance they keep, the stronger interaction they have [20]. The interaction between Ni species and HZSM-5 can be proved by XRD and TPR analysis. In different states, the difference of the interaction causes significant diversity of desulfurization performance. Active Ni species of RA catalyst that filling in the coupling-bed are introduced by impregnation, so Ni species can disperse well after calcination, which can contact closely with HZSM-5 to form an interactive system in atomic level. According to Fig. 6, the desulfurization performance of RA catalyst has a great difference with the others, it can be caused by strong interaction between Ni species and ZSM-5 that forms through the certain preparing process. Preparing method of RA catalyst determines that Ni species can be uniformly deposited on HZSM-5, the sticking probability is relatively high. During the calcination process, both Ni species and HZSM-5 will obtain energy to be activated so that Ni species may diffuse into pore or even framework of the HZSM-5 (as shown in Fig. 3) under the driving of heating. When Ni species establish a strong interaction system with HZSM-5, mass and energy transfer between them will be much easier and faster, so that the desulfurization performance of HZSM-5 can be improved.

In contrast, the interaction between Ni species and HZSM-5 of catalysts filling in the mixing-bed or separating-bed just belongs to macroscopical contact or adjacency. There is not a very strong force between two active species in these two states, so the interaction is very weak, even no so-called interaction forms to enhance the desulfurization performance of catalytic system. Although Ni species and HZSM-5 are all in the catalytic system, the distance between them makes it difficult to deliver mass and energy efficiently, which makes the reactions in mixing-bed and separating-bed be slowed down by the poor connection compared with that performs in the coupling-bed. So, the reaction in the coupling-bed had the best desulfurization performance, and the worst one was separating-bed. With the whole reaction in mixing-bed and separating-bed carries through, Ni species and HZSM-5 work as two lonely parts to fight against the sulfides, respectively. Both of them have a heavy burden, so most of desulfurization active sites encounter inactivation because of excessive vulcanization or adsorption saturation, which makes the desulfurization rate drop quickly as shown in Fig. 6.

**Fig. 7:**
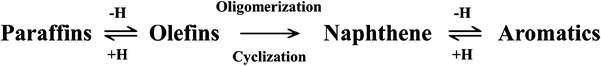
Scheme of transformation between components of FCC gasoline in catalytic systems

Aromatization performance is valued by content change of olefins and aromatics in different products. Olefin content in product of RA catalyst in coupling-bed has a significant decrease as shown in Table 3, and the content of other components all rise in different degrees. In mixing-bed and separating-bed, not only olefin content decreases, paraffins also decline, while the leftover components rise. According to some researches [21, 22], aromatization of FCC gasoline consists of several steps, which includes cracking, oligomerization, isomerization, cyclization, dehydrogenation, etc. They can be summarized in a simple scheme as shown in Fig. 7. Olefins can transform to aromatics as well as paraffins, and the reactions, which depend on certain catalytic system, are mostly reversible.

**Table 3:** PONA analysis data of products over different bed-filling types (wt%)

Bed-filling types	nP	iP	O	N	A	BZ
Feed	7.09	33.52	30.36	6.52	21.71	0.48
Coupling-bed	7.77	35.06	23.86	8.04	24.71	0.51
Mixing-bed	6.67	33.12	28.50	7.26	23.52	0.49
Separating-bed	6.48	33.05	28.43	7.28	23.95	0.49

*nP* n-paraffins, *iP* i-paraffins, *O* olefins, *N* naphthene, *A* aromatics, *BZ* benzene

In terms of the preparing method of RA catalyst, Ni species and HZSM-5 can contact closely to each other to establish several interactive couples, which may increase the transfer ability between them. In this catalytic system, olefins can adsorb on the surface of catalyst, and get in touch with the interactive couples. The probability of attraction between olefins and HZSM-5 can be increased by the existence of Ni species. Olefin molecules will take part in cracking and oligomerization process due to the strong acidity of HZSM-5, and the equilibrium of aromatization will shift towards positive direction. On the other hand, HZSM-5 can improve the hydrogenation activity of Ni species to increase the saturation of olefins. The transformation from olefins to paraffins can be accelerated, too. Therefore, the reaction will tend to generate more aromatics, and the percentage of paraffins also rises, as the data shown in Table 3.

Table 4 gives the boiling range data of products over different bed-filling types. The data show that boiling range of product of all kinds of types decrease, which could be attributed to the cracking function of HZSM-5.

**Table 4:** Boiling range data of products over different bed-filling types (°C)

	Coupling-bed	Mixing-bed	Separating-bed
IBP/10 %	17.7/41.6	19.6/43.0	19.8/42.8
20 %/30 %	54.3/69.4	56.3/71.7	56.6/71.9
50 %	108.5	110.0	110.3
70 %/80 %	145.3/160.6	147.5/162.9	148.0/163.2
90 %/FBP	179.5/211.3	181.6/211.8	181.5/211.7

In comparison with the RA catalyst, increased distance between Ni species and HZSM-5 in mixing-bed and separating-bed leads to decrease of the interaction between them. The content of aromatics in the products of mixing-bed and separating-bed only have a little rise for the significant decrease of catalytic performance. Hydrogenation activity of Ni species also becomes weaker than that is in coupling-bed, which makes a decline of paraffins coming from saturation of olefins. However, HZSM-5 can still catalyze the cracking of paraffins to a certain extent. Therefore, aromatization of olefins performs badly, and the percentage of paraffins in products drops down.

**Fig. 8:**
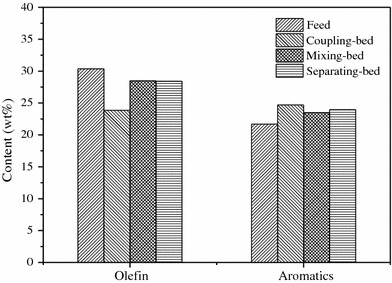
Content of olefins and aromatics in products over different bed-filling types

To further investigate the influence of aromatization on the recovery of octane number (RON) in different bed-filling types, the contents of olefins and aromatics in the products are compared specially. Figure 8 shows the change of olefins and aromatics in FCC gasoline before and after treatment under different states. It can be seen that RA catalyst in coupling-bed exhibits better performance than others. The content of olefins decreases by 6.5 %, when the content of aromatics increases by 3.0 %. The octane number (RON) is only 0.3 lower than the feed.

According to the comparison of aromatization performance over these three states, two active components in RA catalyst, which is prepared by impregnation, can disperse well and establish interactive system, which can promote aromatization by enhancing cracking and oligemerization of low carbon number olefins. In addition, efficient reaction lowers the chance of polymerization of olefins, thus carbon deposition results from olefin polymerization on catalyst can be partly inhibited, and the life of catalysts is improved. Although both Ni species and HZSM-5 exist in the system at the same time in mixing-bed and separating-bed, the closely related compound system with interaction is not formed, leading to a poor aromatization performance. A similar change of olefins and aromatics between mixing-bed and separating-bed, indicating that the interaction between two active components which can promote the aromatization is mainly related to their dispersion and combination, rather than macroscopical distance.

## Conclusion

RA catalyst prepared by impregnation shows higher desulfurization and aromatization performance. The main reason is that Ni species can be well dispersed on HZSM-5 by impregnation, and close relationship can be established between them after calcination, leading to the formation of compound system with interaction. The existence of Ni species promotes the break of C–S bond on acid sites of HZSM-5 during the process of reactive adsorption desulfurization, and it also enhances the cracking and oligomerization of olefins, which makes the equilibrium of aromatization shift towards positive direction and slows the deactivation caused by carbon deposition.
